# 46‐kg abdominal tumor misdiagnosed as obesity: Unveiling healthcare bias due to obesity stigma

**DOI:** 10.1002/ccr3.9360

**Published:** 2024-11-01

**Authors:** Giacomo Calini, Matteo Rottoli, Antonietta D'Errico, Gilberto Poggioli

**Affiliations:** ^1^ Surgery of the Alimentary Tract IRCCS Azienda Ospedaliero‐Universitaria di Bologna Bologna Italy; ^2^ Department of Medical and Surgical Sciences (DIMEC) Alma Mater Studiorum University of Bologna Bologna Italy; ^3^ Pathology Unit IRCCS Azienda Ospedaliero‐Universitaria di Bologna Bologna Italy

**Keywords:** case report, obesity, ovarian tumor, stigma

## Abstract

**Key Clinical Message:**

Obesity results in higher risk of some cancers while obesity stigma affect patient's quality of care. In this case report, a 46 kg ovary mass was misdiagnosed as severe obesity. Obesity stigma awareness and a sustained effort from healthcare professionals are required to deliver adequate patient care to patients with obesity.

**Abstract:**

Obesity is a disease associated with an increased risk of cardiovascular diseases, diabetes, musculoskeletal disorders, and some cancers. Obesity stigma affect patients and healthcare professionals leading to mistrust, poor adherence, noncompliance to screening, and misdiagnosis. We reported a case report of a patient sent to our referral center for surgical evaluation of long‐standing severe obesity (BMI 59). Physical examination was significant for abdominal obesity with a hard consistency, but no cushingoid dysmorphism or lipodystrophy. No abdominal pain, pelvic pain, vaginal bleeding, or change in bowel movements were present. Tumor markers were normal except for an elevated Ca 19.9. Imaging showed a large, intraperitoneal abdominal mass with no metastatic disease. The patient underwent surgery to remove a 46‐kg complex ovarian cystic mass (circumference: 160 cm, diameter: 67 cm), full of liquid and with six nodular areas. The mass was entirely extracted with an intact capsule. The cystic mass resulted in a well‐differentiated intestinal‐type adenocarcinoma with microinvasive foci, an endophytic borderline area (sec. WHO 2014), and mucinous‐cystic areas with no atypia. The patient had postoperative bilateral basal pleural effusion resolved with conservative treatment and was discharged at home on postoperative day 12 with an uneventful 90‐day postoperative follow‐up. In the present case report, a 46 kg ovary mass was misdiagnosed as severe obesity, and the patient was referred for bariatric evaluation. Unveiling biases related to obesity stigma is the first step to ensuring better patient care. Obesity stigma awareness and a sustained effort from healthcare professionals are required to deliver adequate patient care to patients with obesity.

## 1 INTRODUCTION

Obesity is a complex disease defined as abnormal or excessive fat tissue accumulation that presents a risk to health.^1^ According to the World Health Organization (WHO), obesity is a latent disease declared as a noninfectious and noncommunicable pandemic.^1^, ^2^ Indeed, obesity is linked to more deaths worldwide than being underweight, being a risk factor for cardiovascular diseases, diabetes, musculoskeletal disorders, and some cancers.^1^, ^2^ In particular, obesity is associated with 13 different cancers, among them: breast, endometrial, ovarian, prostate, liver, gallbladder, kidney, and colon and rectum^3^, ^4^ Most frequently, obesity is a primary condition, while differential diagnosis of secondary obesity includes: hypothyroidism, hypercortisolism, hyperinsulinism (type 2 diabetes), polycystic ovary syndrome, and medications.

Obesity stigma refers to social devaluation and denigration due to excess body weight.^5^ Several large‐scale studies across various countries showed that a high percentage of patients suffered from obesity stigma also from healthcare professionals.^6^, ^7^ And, obesity stigma has been related to higher risk of misdiagnosis.^6^, ^7^ In particular, primary care physicians spend less time during office visits with patients with obesity as they consider them as noncompliant patients.^8^ Therefore, experiences of and expectations for poor treatment may cause avoidance of care, mistrust of doctors, and poor adherence among patients with obesity.^8^, ^9^ Obesity stigma can reduce the patient's quality of care, including preventive and screening care, despite the best intentions of healthcare professionals to provide high‐quality care.^8^, ^9^, ^10^

The patient was sent to a referral center by the primary care provider for surgical evaluation for long‐standing severe obesity (BMI 59). This case report is unique because of its clinical relevance to obesity differential diagnosis.

## 2 CASE HISTORY

### 2.1 Patient information

The patient was a 53‐year‐old female, she arrived at our center for long‐standing severe obesity (145 kg × 157 cm, BMI 59) after a weight loss of 30 kg in the last 12 months following a hypocaloric diet. The BMI was in the overweight range since adolescence, increased to the obesity range after the first pregnancy (30 yo), and to the severe obesity range after the second pregnancy (34 yo). Comorbidities were type 2 diabetes mellitus, arterial hypertension, hypothyroidism, and multinodular goiter. Therapy at home included: Levotiroxin 75 mcg at 8 am, Irbesartan/Hydrochlorothiazide 300/12.5 mg at 8 am, Metformin 850 mg at breakfast, 1000 mg at lunch, 1000 mg at dinner, and Torasemide 10 mg every other day. Two years before our encounter she underwent endoscopic surgery for an in situ endometrial cancer, but she had not completed follow‐up imaging properly. The screenings for breast (mammography) or colon cancer (fecal occult blood test or colonoscopy) have not been performed. At the time of our encounter, the patient had been in menopause for 3 years. She was allergic to mushrooms and did not report smoking, alcohol, or recreational drug habits. She reported satisfactory sleep, regular intestinal transit, and normal micturition. Level of education was consistent with level 5: short‐cycle tertiary education. Family history was relevant for stroke (living father), arterial hypertension (living mother), and severe obesity (two living brothers).

### 2.2 Clinical findings

Physical examination was significant for abdominal obesity with a hard consistency. The abdomen was globose and distended with abundant intrabdominal and subcutaneous fat tissue with an increased waist‐to‐hip ratio but no cushingoid dysmorphism or lipodystrophy. Long‐standing bilateral leg edema with orange‐peel skin thickening was present, but no abdominal pain, pelvic pain, vaginal bleeding, and change in bowel movements. Cardiac examination was significant for a 2–3/6 systolic murmur best heard at the mitral valve area. No neurological or pulmonary pathological findings at physical examination. The patient walked only with help. Differential diagnoses at this point included: severe obesity, metabolic syndrome, hypercortisolism, abdominal mass, ascites, and intestinal bloating.

## 3 METHODS

### 3.1 Differential diagnosis, investigation, and treatment

**FIGURE 1:**
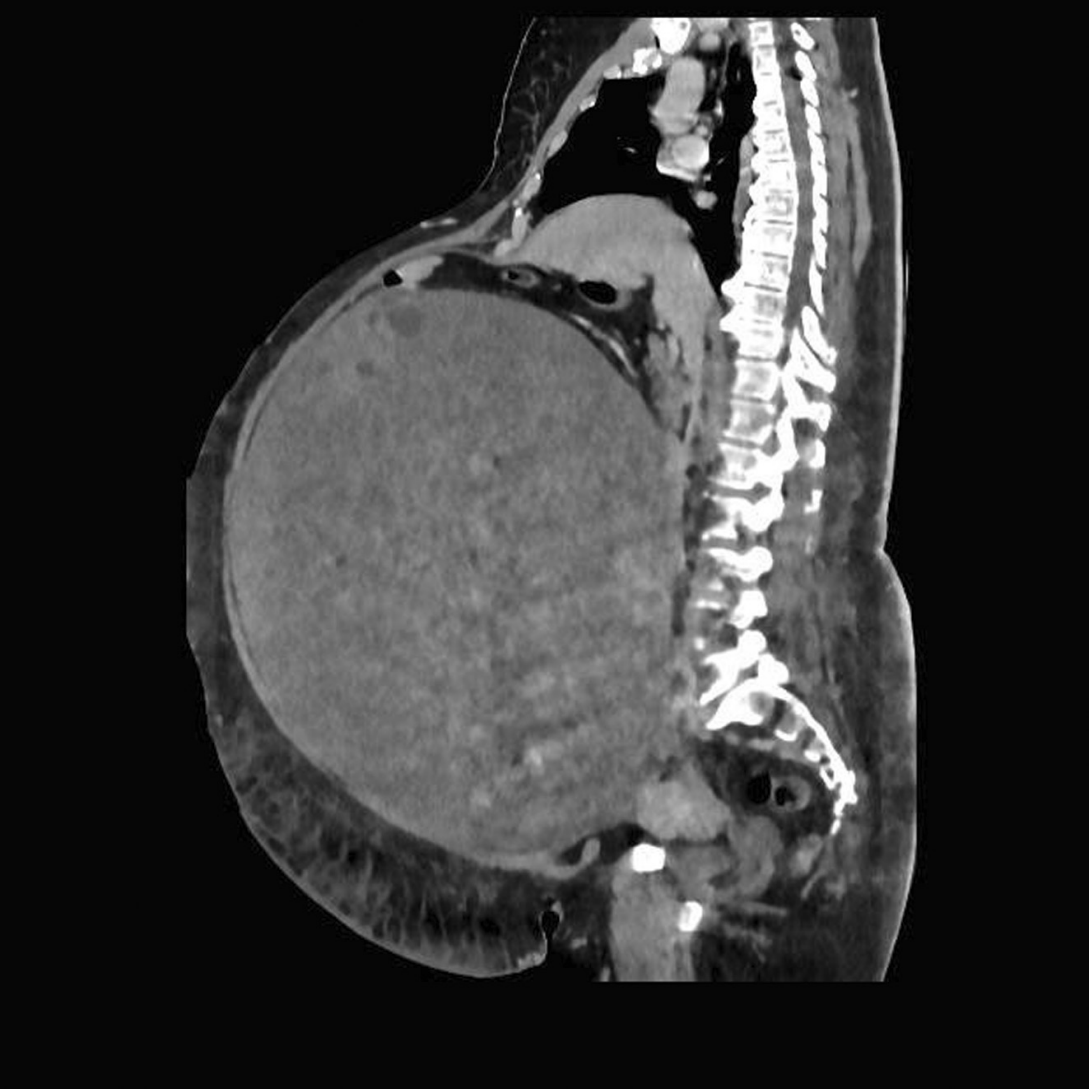
CT scan. Coronal view of the abdominal CT scan.

The patient was admitted to an Internal Medicine ward. Full laboratory testing, legs, abdominal and cardiac ultrasounds, lung function test, and endocrine and surgical bariatric evaluations were requested after the physical examination to explore the major clinical findings (abdominal obesity, cardiac murmur, and leg edema). Laboratory testing found nonspecific results except for elevated ca 19–9 and hyperandrogenism. Carcinoembryonic antigen (CEA), Ca125, Ca15‐3, and alpha‐fetoprotein (AFP) were in range. The endocrinologist evaluated the patient for known diabetes and hypothyroidism and excluded hypercortisolism as the cause of obesity. Hypothyroidism, diabetes mellitus, and arterial hypertension were well treated with current therapy. Among the differentials, ascites and intestinal bloating were excluded according to normal bowel function and incongruent physical examinations. Legs Doppler ultrasound showed continent and normal flow in the superficial and deep venous system with no deep venous thrombosis (DVT), subcutaneous bilateral pretibial and tight edema, and bilateral Baker cyst. Lung function test showed a mild restrictive deficit with no contraindication to surgery. Cardiology evaluation determined a moderate preoperative risk according to left axial deviation at EKG, and cardiac ultrasound results: degenerative valvular alteration, mild mitral insufficiency, and mild ascending aortic dilatation with EF 62%. Physical therapy was performed. Because of the patient's constitution and elevated Ca19‐9, the surgeon suggested performing a CT scan instead of abdominal ultrasound. CT scan revealed a very large, intraperitoneal abdominal mass (40 × 32 cm) with defined margins, mixed density, and partially enhanced by contrast, with no abdominal metastasis, free fluid, or lymphadenopathy, and a single 15 mm gallstone (Figure 1). However, the CT‐scan was not conclusive about the precise origin of the mass. The differential diagnosis included soft tissue sarcomas, ovarian cancer, gastrointestinal stromal tumors, and benign tumors. The abdominal mass failed a CT‐guided biopsy with subsequent C‐reactive protein elevation and low‐grade fever treated with a short course of third‐generation cephalosporin. The biopsy sample was insufficient for pathology. Therefore, the surgical evaluation indicated performing a tumor excision surgery.

## 4 CONCLUSION AND RESULTS

### 4.1 Surgical intervention and postoperative stay

**FIGURE 2:**
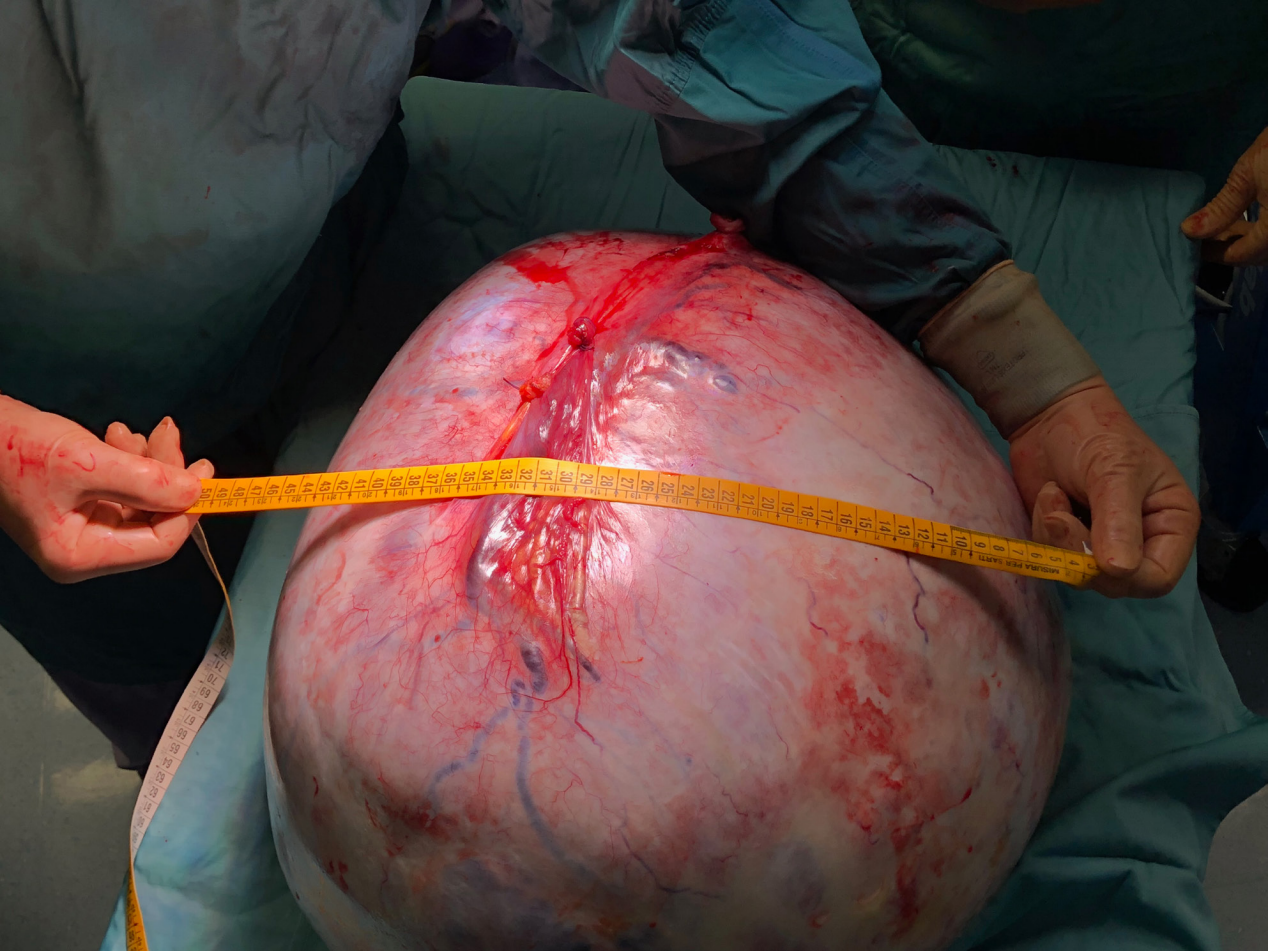
Intraoperative surgical specimens. 46‐kg complex ovarian cystic mass (circumference: 160 cm, diameter: 67 cm).

**FIGURE 3:**
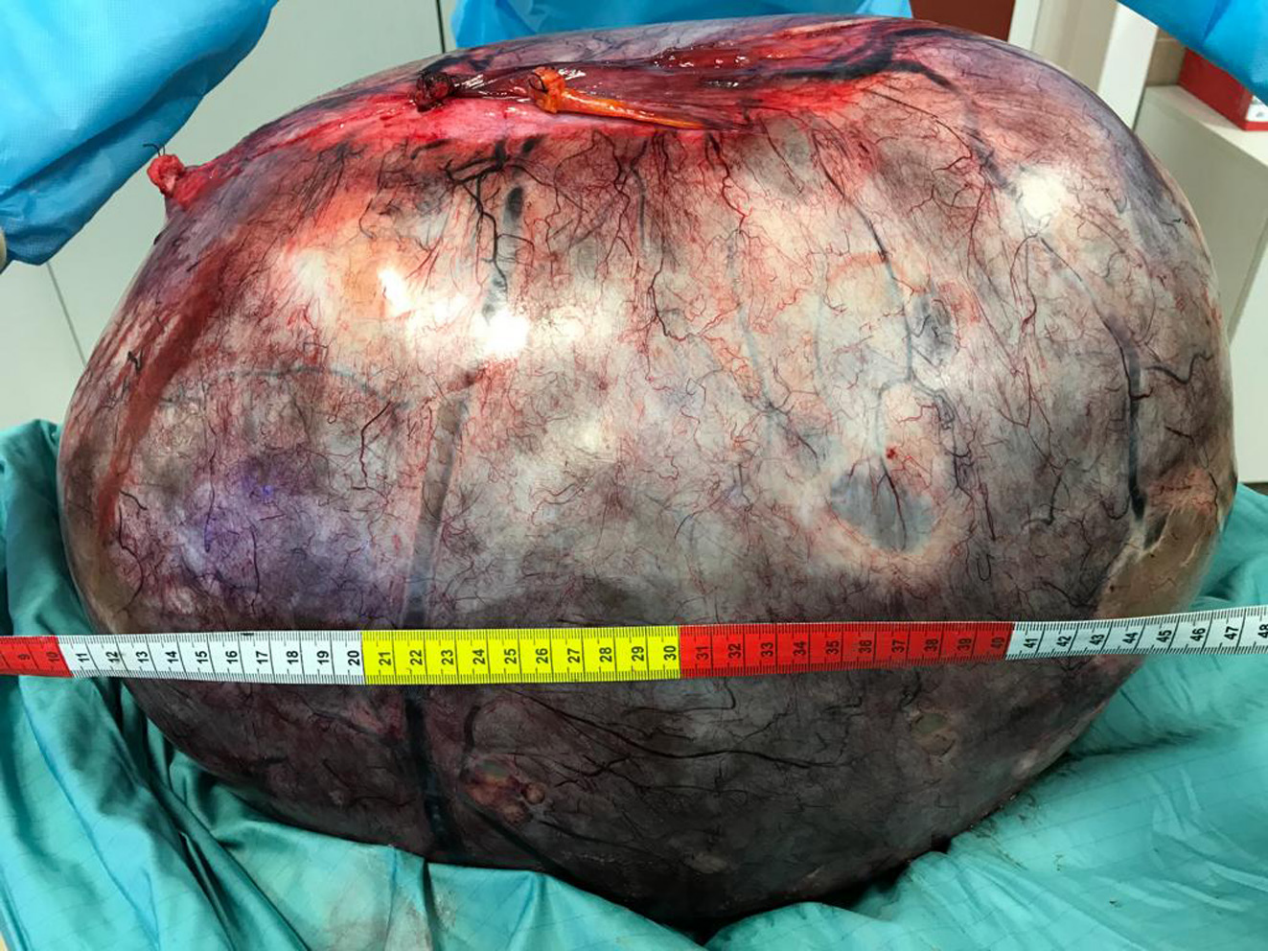
Intraoperative surgical specimens. 46‐kg complex ovarian cystic mass (circumference: 160 cm, diameter: 67 cm).

Surgery was performed under general anesthesia. Preoperatively, a central venous catheter (CVC), nasogastric tube, and urinary catheter were placed. Antibiotic prophylaxis was performed with cefazolin. The abdomen was entered through a xiphopubic incision. Intrabdominal exploration confirmed a very large mixed mass originating from the left ovary with clear tumor margins. A complete excision of the mass, including the left tube and omentum, was performed and sent for pathology (Figures 2 and 3). Considering the long‐standing presence of the abdominal mass and its unknown benign/malignant nature, further surgical procedures were postponed. Therefore, the appendix and contralateral ovary were left in situ. Two intrabdominal drain tubes were placed in the pelvis and the abdomen was closed in layers.

At the end of the surgery, the patient was extubated in the postanesthesia care unit. Intravenous fluid, pain control drugs, and low‐molecular‐weight prophylaxis were given. Micturion without urinary catheter was achieved in the postoperative day (POD) 1, while abdominal tubes started to drain >2000 mL/day of limpid liquid. At POD 2, the patient restarted bowel function and eating progressively. At POD 4, the pulse oximeter showed an 88% oxygen level while the patient was resting supine, which improved with a sitting position, and without any symptoms, a chest x‐ray and arterial blood gas (ABG) test were performed, revealing a bilateral basal pleural effusion with normal ABG. The abdominal tubes' amount of liquid and the pleural effusion were treated with albumin and diuretic infusions until they were removed in POD 6 and POD 10 and the oxygen level returned to >94%. The surgical incision healed well, the postoperative course was uneventful, and the patient was discharged at home on POD 12.

### 4.2 Follow‐up

No postoperative complications were found at the 90‐day follow‐up.

**FIGURE 4:**
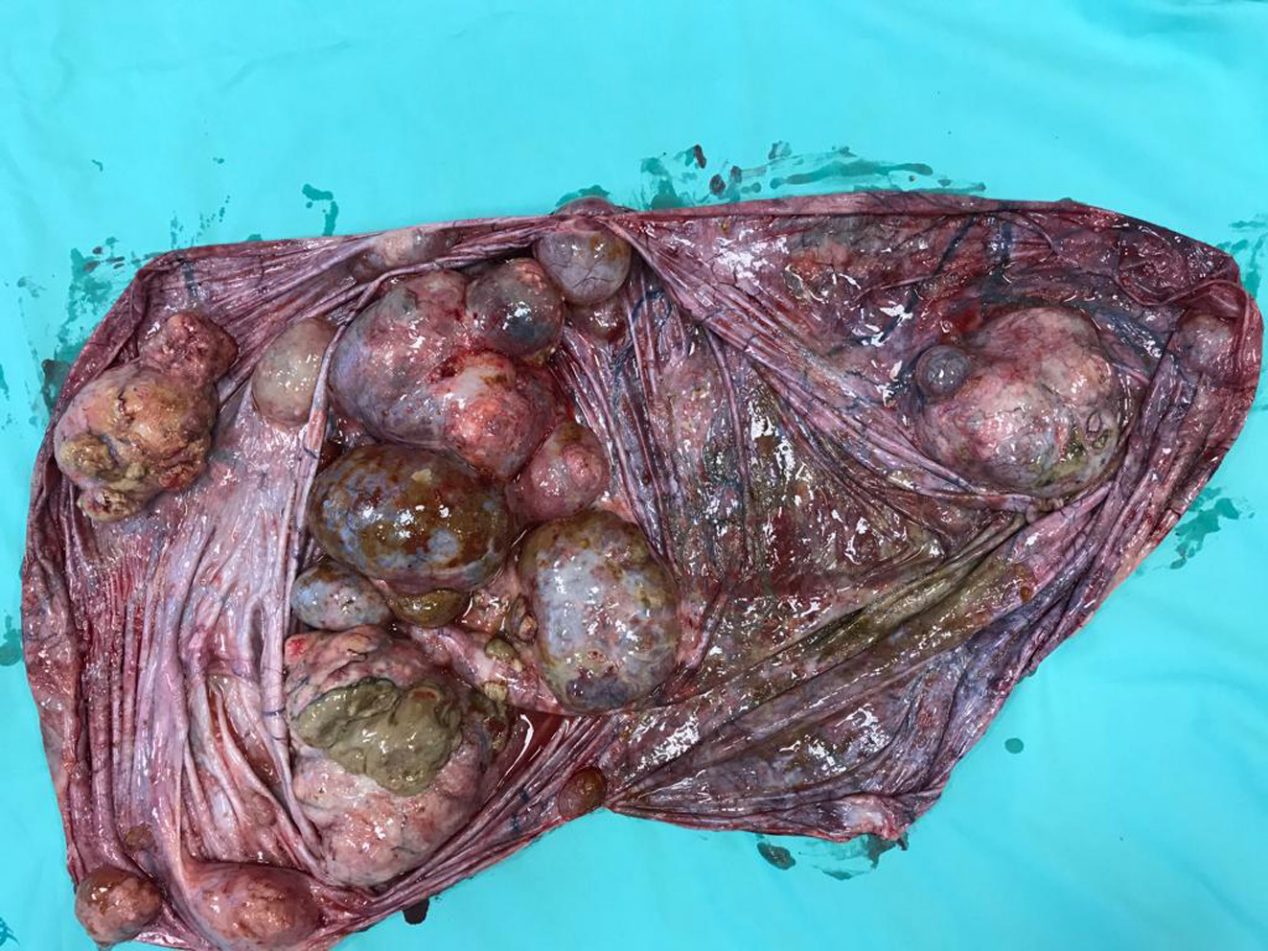
Macroscopic pathology. Six nodular areas detected at macroscopic pathology.

Histology revealed a 46‐kg complex ovarian cystic mass (circumference: 160 cm, diameter: 67 cm), full of brownish liquid (43 kg), with six nodular areas (greatest diameter of 15 cm) entirely extracted with an intact capsule, and the Omentum (33 × 25 cm) (Figure 4).

The cystic mass resulted in a well‐differentiated intestinal‐type mucinous carcinoma with areas of mucinous‐cystic morphology. The different areas were as follows: a well‐differentiated intestinal‐type adenocarcinoma intraepithelial and with expansive‐infiltrative microinvasive foci (<5 mm), endophytic borderline areas (sec. WHO 2014) with predominant intestinal differentiation, and mucinous cystic areas with no epithelial atypia. The left fallopian tube and omentum were inflamed but free from tumor disease.

After surgery, the final weight of the patient without the mass was 99 kg (BMI 40). Per the patient's preference, further endoscopic procedures and surgeries (including bariatric surgery) were deferred.

## 5 DISCUSSION

Obesity is strongly associated with numerous chronic diseases such as type 2 diabetes, cardiovascular diseases, dyslipidemia, nonalcoholic fatty liver disease, chronic kidney disease, obstructive sleep apnea, infection, mood disorders, and physical disabilities.^11^ In addition, a relation between obesity and increased cancer risk has been demonstrated.^3^, ^4^, ^11^, ^12^ In particular, obesity is a well‐known risk factor for ovarian cancer.^13^, ^14^

In the described case report, a gigantic abdominal mass (46 kg; 40 × 32 cm) was misdiagnosed as severe obesity (BMI 59). Indeed, the patient's factual obesity (BMI 40 without the mass weight), a positive family history, type 2 diabetes, and hypothyroidism might have influenced primary care and hospital care personnel. However, a rigid abdominal mass at physical examination should raise concern about its neoplastic nature even in a patient with severe obesity. Based on the literature, the finding of an abdominal mass with similar characteristics is rare in developed countries where a universal public healthcare system is available.^15^ Indeed, most of the giant masses reported in literature come from least developed or developing countries where the lack of accessibility to healthcare explain the diagnosis and treatment delays.^12^, ^15^ Differently, in this case report, the misdiagnosis seems based on the stigma towards obesity, which reduced patient screening adherence, affected primary care visits, and delayed the request for further investigations of the clinical presentation of the patient.^8^, ^9^, ^10^ Indeed, the patient missed screening for breast and colon cancer and did not completed proper follow‐up imaging after the endoscopic resection of an in situ endometrial cancer. In addition, abdominal obesity with hard consistency should raise suspect of secondary obesity (e.g., Cushing disease and abdominal mass) that was not rule out before sending her to a referral center for bariatric surgery. Similarly, atypical long standing leg edema were not investigated with legs Doppler ultrasound prior to the hospital admission.

As discussed, obesity is related to an increased risk of associated diseases (e.g., cardiovascular diseases, diabetes, musculoskeletal disorders, and cancers). Obesity‐related complications are common, increase healthcare resource use, and are associated with lower quality of life, and higher mortality.^16^ Nevertheless, obesity stigma remains abundant throughout entertainment, social media, advertising, news outlets, and political and healthcare landscapes.^9^ This has psychological, physical, and socioeconomic consequences and does not prevent obesity, while, the obesity stigma is (un‐)consciously present throughout society affecting also healthcare personnel.^9^

A critical analysis of the misdiagnosis reported in the present case reports and its relation to obesity stigma needs to consider several circumstances, clinical decisions, and biases.

As a result of large‐scale studies across various countries, almost half of the patients with obesity suffer devaluation due to obesity stigma affecting healthcare professionals.^6^, ^7^ Primary care physicians spend less time during office visits with patients with obesity as they consider them noncompliant patients.^8^ Indeed, the patients had long‐standing abdominal obesity with a hard consistency that was not investigated further. However, hypothyroidism, diabetes mellitus, and arterial hypertension were well treated with the therapy at home. Therefore, it is unlikely that poor care was the reason for the misdiagnosis.

Also, experiences of and expectations for poor treatment may cause avoidance of care, mistrust of doctors, and poor adherence among patients with obesity.^8^, ^9^ Indeed, abdominal imaging has been planned as a follow‐up after she underwent endoscopic surgery for an in situ endometrial cancer. However, she was not able to enter the CT scan machine at her hometown local hospital due to the size of her abdomen. The following diagnostic imaging in a suitable CT scan machine was not replanned. In addition, after the pathological diagnosis of the tumor mass, the patient declined further endoscopic and surgical procedures. As previously reported, obesity stigma can reduce the patient's quality of and adherence to care, including preventive and screening care, despite the best intentions of healthcare professionals to provide high‐quality care.^8^, ^9^, ^10^

There are several potential strategies to address obesity stigma in healthcare.

It is pivotal to raise awareness about obesity stigma affecting healthcare professionals. In the primary care setting it is important to improve attitudes about patients with obesity: reducing the likelihood of negative attitudes, altering the clinic environment or procedures to create a setting where patients with obesity feel accepted, and empowering patients to cope with stigmatizing situations and attain high‐quality health care.^8^

Obesity should be treated as a disease itself and patients with obesity should not be considered noncompliant patients. Taking care of patients with obesity includes also evaluating and screening them for the increased risk of associated diseases (e.g., cardiovascular diseases, diabetes, musculoskeletal disorders, and cancers). Also, reducing the focus on body weight while encouraging feasible behaviors will improve health and well‐being.^8^

Obesity stigma should also be addressed on a clinical environment level, conveying a sense of identity safety by providing evidence that diversity is valued. Possible solutions are providing medical equipment and chairs that are usable by patients of all sizes and keeping specialized instruments for patients with obesity readily available to clinical staff.^8^

Major points to prevent this situation in future are raising awareness among primary care physicians and patients with obesity of the higher risk of cancer and the reduced preventing and screening compliance in patients with obesity. Along with these healthcare‐specific interventions, further political, sociocultural, psychological, social media, and entertainment industry interventions are needed.^9^

## 6 CONCLUSION

In the present case report, a 46 kg ovary mass was misdiagnosed as long‐standing severe obesity, and the patient was referred for bariatric evaluation. Unveiling biases related to obesity stigma is the first step to ensure better patient care. Obesity stigma awareness and a sustained effort from healthcare professionals are required to deliver adequate patient care to patients with obesity.

## AUTHOR CONTRIBUTIONS

**Giacomo Calini:** Conceptualization; data curation; writing – original draft. **Matteo Rottoli:** Conceptualization; data curation; investigation; methodology. **Antonietta D'Errico:** Conceptualization; investigation. **Gilberto Poggioli:** Conceptualization; data curation; project administration; resources.

## FUNDING INFORMATION

Financial support for open access was provided by funds of the Department of Medical and Surgical Sciences (DIMEC), Alma Mater Studiorum University of Bologna, Bologna, Italy.

## CONFLICT OF INTEREST STATEMENT

The authors declare no conflict of interest.

## CONSENT

Written informed consent was obtained from the patient to publish this report in accordance with the journal's patient consent policy.

## Data Availability

Data are fully presented in this manuscript. Further underlying information will be shared on reasonable request to the corresponding author and according to the patient's identity protection and consent.
